# Acceptance of the coronavirus disease-2019 vaccine among medical students in Uganda

**DOI:** 10.1186/s41182-021-00331-1

**Published:** 2021-05-13

**Authors:** Andrew Marvin Kanyike, Ronald Olum, Jonathan Kajjimu, Daniel Ojilong, Gabriel Madut Akech, Dianah Rhoda Nassozi, Drake Agira, Nicholas Kisaakye Wamala, Asaph Asiimwe, Dissan Matovu, Ann Babra Nakimuli, Musilim Lyavala, Patricia Kulwenza, Joshua Kiwumulo, Felix Bongomin

**Affiliations:** 1 Faculty of Health Sciences, Busitema University, Mbale, Uganda; 2 School of Medicine, College of Health Sciences, Makerere University, Kampala, Uganda; 3 Faculty of Medicine, Mbarara University of Science and Technology, Mbarara, Uganda; 4 School of Health Sciences, College of Health Sciences, Makerere University, Kampala, Uganda; 5 School of Medicine, Kabale University, Kabale, Uganda; 6 Faculty of Clinical Medicine and Dentistry, Kampala International University, Ishaka-, Bushenyi, Uganda; 7 School of Health Sciences, Soroti University, Soroti, Uganda; 8 Faculty of Biology, Medicine and Health, King Ceaser University, Kampala, Uganda; 9 School of Health Sciences, Uganda Christian University, Mukono, Uganda; 10 Faculty of Medicine, Islamic University in Uganda, Mbale, Uganda; 11 Faculty of Medicine, Gulu University, Gulu, Uganda; 12 Department of Medical Microbiology and Immunology, Faculty of Medicine, Gulu University, Gulu, Uganda; 13 Department of Medicine, College of Health Sciences, Makerere University, Kampala, Uganda

**Keywords:** COVID-19, Vaccine acceptance, Vaccine hesitancy, Medical students

## Abstract

**Background:**

COVID-19 is still a major global threat for which vaccination remains the ultimate solution. Uganda reported 40,751 cases and 335 deaths as of 9 April 2021 and started its vaccination program among priority groups like health workers, teachers, those with chronic diseases among others in early March 2021. Unanimous uptake of the COVID-19 vaccine is required to subsequently avert its spread; therefore, we assessed COVID-19 vaccine acceptability, hesitancy, and associated factors among medical students in Uganda.

**Methods:**

This study employed an online descriptive cross-sectional survey among medical students across 10 medical schools in Uganda. A structured questionnaire via Google Form was conveniently sent to eligible participants via WhatsApp. Each medical school had a coordinator who consistently shared the data tool in the WhatsApp groups. Chi-square or Fisher’s exact test, and logistic regression were used to assess the association between vaccine acceptability with demographics, COVID-19 risk perception, and vaccine hesitancy.

**Results:**

We surveyed 600 medical students, 377 (62.8%) were male. COVID-19 vaccine acceptability was 37.3% and vaccine hesitancy 30.7%. Factors associated with vaccine acceptability were being male (adjusted odds ratio (aOR) = 1.9, 95% CI 1.3–2.9, *p*=0.001) and being single (aOR= 2.1, 95% CI 1.1–3.9, *p*=0.022). Very high (aOR= 3.5, 95% CI 1.7–6.9, *p*<0.001) or moderate (aOR =2.2, 95% CI 1.2–4.1, *p*=0.008) perceived risk of getting COVID-19 in the future, receiving any vaccine in the past 5 years (aOR= 1.6, 95% CI 1.1–2.5, *p*=0.017), and COVID-19 vaccine hesitancy (aOR 0.6, 95% CI 0.4–0.9, *p*=0.036).

**Conclusions:**

This study revealed low levels of acceptance towards the COVID-19 vaccine among medical students, low self-perceived risks of COVID-19, and many had relied on social media that provided them with negative information. This poses an evident risk on the battle towards COVID-19 in the future especially when these future health professions are expected to be influencing decisions of the general public towards the same.

## Introduction

The coronavirus disease-2019 (COVID-19) pandemic, caused by the novel severe acute respiratory syndrome-coronavirus-2 (SARS-CoV-2) continues to create mayhem across the globe. COVID-19 has affected livelihoods and has imposed strains on the health care systems [1]. Over135 million people have been infected with SARS-CoV-2 resulting in over 2.9 million deaths worldwide [2]. The African continent has continuously recorded fewer cases of COVID-19 with about 4.3 million cases and 115,321 deaths [2]. Uganda reported 40,751 cases with 335 deaths as of 9 April 2021 [3].

Various chemotherapeutic and biologic therapies, like hydroxychloroquine, remdesivir, convalescent plasma, and tocilizumab, have been tried to treat COVID-19 patients [4–6] with no conclusive specific curative effect. Different preventive public health measures like lockdowns, hand washing, respiratory hygiene, and social distancing have been employed with little success [7]. Much worse even, attempts to loosen these precautionary behaviors have culminated in the surge of cases in many countries [7]. Leaving room for only an effective vaccine as a long-lasting solution in such a crisis [8, 9].

Several vaccine candidates have been developed to date with some approved and others still undergoing clinical trials. Notably, the New York-based Pfizer-BioNTech, ModernaInc company, and the AstraZeneca/University of Oxford Vaccines have been approved for emergency use and already rolled out in some countries including Uganda [10]. Although much progress has been made with vaccine development, uncertainty about the public acceptance of COVID-19 vaccination is still an important challenge [9]. The World Health Organization (WHO) asserts that vaccine hesitancy is one of the top ten threats to global health and this is exacerbated by the emerging conspiracies surrounding COVID-19 and its vaccines [11].

Medical students are regarded as an insightful population that is open-minded, educated, and medically informed. They also represent the future health professionals, who are supposed to respond quickly to public health issues [12]. Surprisingly, a study done in the USA reported that nearly one-quarter of the medical students were hesitant to be vaccinated as soon as an approved COVID-19 vaccine becomes available, despite self-perception of elevated risk of exposure to SARS-CoV-2 infection [13]. Furthermore, another study done in Israel reported a high rate of COVID-19 vaccine skepticism among medical staff implying that vaccination compliance, even among medically informed individuals, is not automatic [14].

The Ugandan government through the COVAX facility received its first 864,000 doses of the AstraZeneca vaccine in early March [15]. Subsequently, Uganda rolled out COVID-19 vaccination across the country, starting with priority groups consisting of healthcare workers, security personnel, teachers, humanitarian frontline workers, and patients at higher risk of severe COVID-19 disease among others [15].

In Uganda, medical students form a core part of the health care response team in regional and national referral hospitals making them a vulnerable group [16]. They are also an important force in health education and communication in their various communities. It is therefore imperative to assess the acceptability and attitudes of these students towards the COVID-19 vaccine. To our knowledge, no such study has been done in Uganda, and Africa at large. Therefore, we aimed to assess COVID-19 vaccine acceptability, hesitancy, and associated factors among medical students in Uganda.

## Methods

### Study design

We conducted an online, descriptive, cross-sectional study between Monday 15 March and Sunday 21 March 2021 using a quantitative approach.

### Study setting

The study was carried out in 10 universities in Uganda offering undergraduate medical degrees, namely, Makerere University (Mak), Mbarara University of Science and Technology (MUST), Gulu University (GU), Kampala International University (KIU), Kabale University (KU), Busitema University (BU), Islamic University in Uganda, Soroti University (SU), King Caesar International University, and Uganda Christian University (UCU). Mak, GU, MUST, BU, KU, and SU are public universities, and the remaining universities are private. The combined population size of all these medical schools is about 6000–8000 students.

### Study population

Medical students pursuing the following undergraduate degree programs in these various universities were targeted: Bachelor of Medicine and Bachelor of Surgery (MBChB), Bachelor of Dental Surgery (BDS), Bachelor of Nursing (BNS), Bachelor of Anesthesia (BNA), Bachelor of Pharmacy (BPHARM), Bachelor of Biomedical Laboratory Technology (BLT), and Bachelors of Biomedical Sciences (BBS). MBChB and BDS courses run for 5 years; BNS, BNA, and BPHARM are done for 4 years while BLT and BBS go for 3 years in our sampled universities.

### Inclusion and exclusion criteria

Individuals aged 18 years or older, currently, students in the abovementioned universities who consented to participate, were included and those students who could not access the Internet were excluded.

### Sampling procedure and data collection

During this study, Uganda was in a partial lockdown with schools, universities, and institutions partially opened conducting hybrid physical and Open Distance E-Learning. Therefore, we opted to use WhatsApp Messenger (Facebook Inc) for enrolling potential participants based on our previous experience with conducting studies among medical students [16]. We employed convenience sampling where we identified all the existing WhatsApp groups of medical students in the various universities through a coordinator for each specific university. The Google Form link to the questionnaire was then sent to the potential participants via the identified WhatsApp groups.

### Data collection tool

We adapted a validated questionnaire as used by Tamam and colleagues [17] and modified it to suit our study population. The questionnaire was structured into four sections. The first section captured socio-demographic information including age, sex, program of study, university, religion among others. The second section assessed COVID-19 pandemic-related information entailing whether the participant was confirmed to be infected with COVID-19 or thought so, if they knew anyone who was infected and confirmed by laboratory test. It also assessed what they thought was the magnitude of threat COVID-19 posed to them, the entire Uganda, and if they believed to have already acquired immunity against COVID-19. The third section was about acceptance and hesitancy to COVID-19 vaccines where participants were asked if they would take the vaccine when availed and give reason for their answers. It also assessed if participants had been reluctant or refused to take vaccines in the past 5 years. The fourth section assessed the attitude towards the COVID-19 vaccine.

### Study variables

Independent variables were the demographic characteristics including sex, age, education program, religion, residence, education institution, and sources of information on COVID-19 and COVID-19 vaccines and dependent variables were the acceptability, hesitancy, trust, and attitudes towards COVID-19 vaccine.

### Data management analysis

Fully completed questionnaires were extracted from Google Forms and exported to Microsoft Excel 2016 (Microsoft Corporation) for cleaning and coding. The cleaned data was exported to STATA (StataCorp LLC, TX, USA) version 16.0 for analyses. Numerical data was summarized as means (standard deviations) or median (inter-quartile range) for parametric and non-parametric data, respectively. Categorical data was summarized as frequencies and proportions. Associations between independent variables and dependent variables were assessed using the chi-square test or Fisher’s exact test and logistic regression analysis in STATA 16.0 software. A *P*<.05 was considered statistically significant.

## Results

### Socio-demographic characteristics

A total of 600 medical students completed the survey. The majority were male (*n*=377, 62.8%), single (*n*=521, 87.1%), of Anglican religion (*n*=184, 30.7%), pursing MBChB degree (*n*=488, 81.3%), and in their fourth year of study (*n*=157, 26.2%). BU had the highest number of participants (*n*=122, 20.4%); meanwhile, the least (*n*=14, 2.3%) number of participants were from UCU. Table 1 summarizes the socio-demographic characteristics of the participants.

**Table 1:** Demographic characteristics of participants

Demographics	Frequency	%
**Age**		
≤24	367	61.2
>24	233	38.8
**Sex**		
Male	377	62.8
Female	223	37.2
**Marital status**		
Single	521	87.1
Married	74	12.4
Separated	3	0.5
**Religion**		
Anglican	184	30.7
Roman Catholic	166	27.7
Muslim	102	17.0
Pentecostal	90	15.0
Other	35	5.8
SDA	21	3.5
Orthodox	2	0.3
**University of Study**		
Busitema University	122	20.4
Kampala international University	102	17.1
Makerere University	89	14.9
Kabale University	71	11.9
Islamic University in Uganda	62	10.4
Mbarara University of Science and Technology	55	9.2
Gulu University	43	7.2
King Caesar University	20	3.3
Soroti University	20	3.3
Uganda Christian University	14	2.3
**Year of study**		
Year 1	91	15.2
Year 2	81	13.5
Year 3	131	21.8
Year 4	157	26.2
Year 5	140	23.3
**Academic program**		
Bachelor of Biomedical laboratory technology	4	0.7
Bachelor of Biomedical sciences	10	1.7
Bachelors of Anaesthesia	16	2.7
Bachelors of Dental Surgery	11	1.8
Bachelors of Medicine and Surgery	488	81.3
Bachelors of Nursing	58	9.7
Bachelors of Pharmacy	13	2.2

*SDA* Seventh Day Adventists

### Acceptability of COVID-19 vaccine and associated factors among medical students

**Fig. 1:**
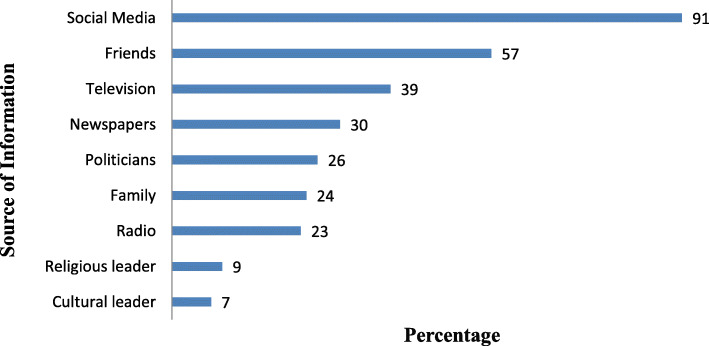
Sources of negative information on the COVID-19 vaccine among medical student

The majority of the participants (*n*=376, 62.7%) were not willing to be vaccinated against COVID-19. The most cited reasons for not taking up the vaccine were concerns about safety (*n*=242, 64.4%) and having heard or read negative information about the vaccine (*n*=201, 53.5%). Of those that reported to have heard negative information about COVID-19 vaccine (*n*=575, 95.8%), the biggest sources were from social media (*n*=521, 90.6%), and friends (*n*=325, 56.5%) (Fig. 1). For the participants willing to take up the COVID-19 vaccine (*n*=224, 37.3%), the major reasons for acceptance were to protect oneself (*n*=191, 85.3%) and others (*n*=142, 63.4%) from COVID-19. Close to half (*n*=111, 49.6%) of the participants believed in vaccines and immunization. Table 2 summarizes reasons for acceptance and hesitancy of the COVID-19 vaccine.

**Table 2:** Acceptability of COVID-19 vaccine among medical students in Uganda

Acceptability of the COVID-19 Vaccine	Frequency	%
**Are you aware of a vaccine for COVID-19?**		
Yes	590	98.3
No	10	1.7
**COVID-19 vaccine may be effective in protecting me from COVID-19.**		
Strongly agree	75	12.5
Agree	198	33.0
Neutral	220	36.7
Disagree	55	9.2
Strongly disagree	52	8.7
**Are you willing to get vaccinated with the approved COVID-19 vaccine?**		
Yes	224	37.3
No	376	62.7
**Reasons for accepting (*n*=224)**		
To protect myself from getting COVID-19.	191	85.3
To protect others from getting COVID-19.	142	63.4
I believe in vaccines and immunization.	111	49.6
To get rid of the virus and end the pandemic.	82	36.6
Health workers’ recommendations.	78	34.8
To be able to travel.	67	29.9
It is a social and moral responsibility.	60	26.8
If the vaccine is free of charge.	58	25.9
If it is available to me.	55	24.6
The vaccines are effective.	47	21.0
The vaccines are safe.	45	20.1
Government recommendations.	40	17.9
Job requirement.	33	14.7
I am at high risk of severe disease.	28	12.5
**Reason for not accepting the vaccine (*n*=376)**		
I don’t think the vaccine is safe/concerned about side effects	242	64.4
I have heard or read negative information on the vaccine.	201	53.5
I don’t think the vaccine is effective	136	36.2
I trust my immunity	106	28.2
I don’t think it is needed	81	21.5
Someone else told me that the vaccine is not safe	68	18.1
I don’t know where to get good/reliable information	57	15.2
Fear of needles	20	5.3
Religious reasons	11	2.9
I don’t know where to get vaccination	8	2.1
Someone else told me they/their child had a bad reaction	8	2.1
Had a bad experience or reaction with previous vaccination	6	1.6
Vaccine development was rushed	6	1.6
Had a bad experience with previous vaccinator/health clinic	4	1.1
Not possible to leave other work (at home or other)	3	0.8
Other beliefs/traditional medicine	2	0.5

**Fig. 2:**
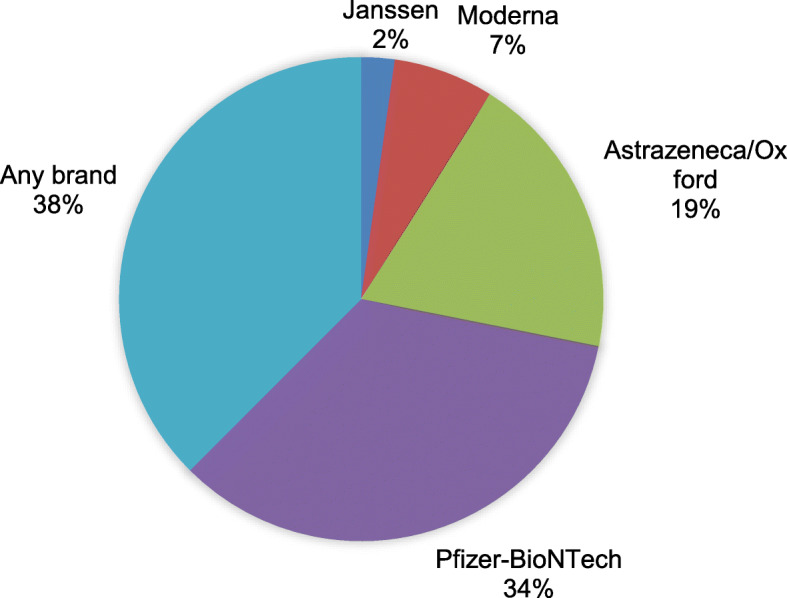
COVID-19 vaccine brand preference among medical students in Uganda (*N*=224)

Of the 224 participants willing to be vaccinated, the majority (*n*=84, 38%) were indifferent to the particular vaccine they would take, 34% (*n*=77) would wish to take the Pfizer-BioNTech vaccine, and only 19% the AstraZeneca vaccine (Fig. 2).

On bivariate analysis, sex (*p*=0.001), belief of getting COVID-19 in the future (*p*<0.001) or having already had it (*p*<0.029), perceived risk of COVID-19 to an individual (*p*=0.001) and Uganda at large (*p*<0.001), belief on effectiveness of the vaccine (*p*<0.001), vaccination uptake in the previous five years (*p*=0.028) and reluctance or hesitancy to vaccination (*p*=0.004) were significantly associated with acceptability of COVID-19 vaccine (Table 3).

**Table 3:** Factors associated with acceptability of the COVID-19 vaccine among medical students in Uganda

Variables	Acceptability		
	No (***N***=376)	Yes (***N***=224)	***p***-value
**Age**			
≤24	224 (61)	143 (39)	0.300
>24	152 (65.2)	81 (34.8)	
**Sex**			
Female	159 (71.3)	64 (28.7)	0.001
Male	217 (57.6)	160 (42.4)	
**Marital status**			
Married	54 (73)	20 (27)	0.124
Separated	2 (66.7)	1 (33.3)	
Single	319 (61.2)	202 (38.8)	
**Religion**			
Anglican	108 (58.7)	76 (41.3)	0.036
Muslim	60 (58.8)	42 (41.2)	
Orthodox	2 (100)	0 (0)	
Other	21 (60)	14 (40)	
Pentecostal	68 (75.6)	22 (24.4)	
Roman Catholic	108 (65.1)	58 (34.9)	
SDA	9 (42.9)	12 (57.1)	
**University of Study**			
Busitema University	83 (68)	39 (32)	0.443
Gulu University	25 (58.1)	18 (41.9)	
Islamic University	41 (66.1)	21 (33.9)	
Kabale University	43 (60.6)	28 (39.4)	
Kampala International University	61 (59.8)	41 (40.2)	
King Caesar University	11 (55)	9 (45)	
Makerere University	53 (59.6)	36 (40.4)	
Mbarara University of Science and Technology	33 (60)	22 (40)	
Soroti University	17 (85)	3 (15)	
Uganda Christian University	7 (50)	7 (50)	
**Year of study**			
Year 1	66 (72.5)	25 (27.5)	0.234
Year 2	53 (65.4)	28 (34.6)	
Year 3	79 (60.3)	52 (39.7)	
Year 4	92 (58.6)	65 (41.4)	
Year 5	86 (61.4)	54 (38.6)	
**Academic Program**			
Bachelor of Biomedical laboratory technology	2 (50)	2 (50)	0.339
Bachelor of Biomedical sciences	3 (30)	7 (70)	
Bachelors of Anaesthesia	9 (56.3)	7 (43.8)	
Bachelors of Dental Surgery	6 (54.5)	5 (45.5)	
Bachelors of Medicine and Surgery	307 (62.9)	181 (37.1)	
Bachelors of Nursing	40 (69)	18 (31)	
Bachelors of Pharmacy	9 (69.2)	4 (30.8)	
**How likely do you think you will get COVID-19 in future?**			
Extremely likely	13 (56.5)	10 (43.5)	<0.001
Moderate	81 (54.7)	67 (45.3)	
Not at all	122 (76.3)	38 (23.8)	
Slightly	122 (66.7)	61 (33.3)	
Very likely	38 (44.2)	48 (55.8)	
**Overall, how worried are you about coronavirus?**			
Extremely	18 (48.6)	19 (51.4)	<0.001
Not at all	69 (75)	23 (25)	
Not very	145 (70.4)	61 (29.6)	
Somewhat	106 (55.8)	84 (44.2)	
Very	38 (50.7)	37 (49.3)	
**To what extent do you think coronavirus poses a risk to you personally?**			
Major risk	71 (53.8)	61 (46.2)	0.001
Minor risk	151 (71.2)	61 (28.8)	
Moderate risk	120 (56.9)	91 (43.1)	
No risk at all	34 (75.6)	11 (24.4)	
**Do you think coronavirus poses a risk to people in Uganda?**			
Major risk	113 (50.2)	112 (49.8)	<0.001
Minor risk	91 (77.1)	27 (22.9)	
Moderate risk	158 (65.8)	82 (34.2)	
No risk at all	14 (82.4)	3 (17.6)	
**Do you know if you have had, or currently have, coronavirus?**			
I have definitely had it	30 (65.2)	16 (34.8)	0.029
I have definitely not had it	134 (65)	72 (35)	
I think I have probably had it	129 (67.5)	62 (32.5)	
I think I have probably not had it	83 (52.9)	74 (47.1)	
**Have you been tested for coronavirus?**			
No	268 (62.5)	161 (37.5)	0.948
Yes—positive	19 (65.5)	10 (34.5)	
Yes—negative	89 (62.7)	53 (37.3)	
**Has any of your family members tested for COVID-19?**			
No	213 (61.7)	132 (38.3)	0.818
Yes—positive	51 (65.4)	27 (34.6)	
Yes—negative	112 (63.3)	65 (36.7)	
**Has any of your friends tested positive for COVID-19?**			
No	156 (61.7)	97 (38.3)	0.729
Yes—positive	143 (64.7)	78 (35.3)	
Yes—negative	77 (61.1)	49 (38.9)	
**I think I have some immunity to coronavirus**			
Agree	110 (59.8)	74 (40.2)	0.088
Disagree	34 (55.7)	27 (44.3)	
Neutral	110 (64.3)	61 (35.7)	
Strongly agree	111 (69.4)	49 (30.6)	
Strongly disagree	11 (45.8)	13 (54.2)	
**Have you been vaccinated before in the past 5 years?**			
No	137 (68.8)	62 (31.2)	0.028
Yes	239 (59.6)	162 (40.4)	
**Have you ever been reluctant or hesitate to get a vaccination before?**			
No	245 (58.9)	171 (41.1)	0.004
Yes	131 (71.2)	53 (28.8)	
**COVID-19 vaccine may be effective in protecting me from COVID-19.**			
Agree	84 (42.4)	114 (57.6)	<0.001
Disagree	53 (96.4)	2 (3.6)	
Neutral	171 (77.7)	49 (22.3)	
Strongly agree	16 (21.3)	59 (78.7)	
Strongly disagree	52 (100)	0 (0)	
**Have you ever received or heard negative information about COVID-19 vaccination?**			
No	17 (68)	8 (32)	0.573
Yes	359 (62.4)	216 (37.6)	

On multivariable logistic regression analysis, significant factors for acceptability were being male (adjusted odds ratio (aOR) = 1.9, 95% CI 1.3–2.9, *p*=0.001), being single (aOR= 2.1, 95% CI 1.1–3.9, *p*=0.022), moderate (aOR=2.2, 95% CI 1.2–4.1, *p*=0.008) or very high (aOR= 3.5, 95% CI 1.7–6.9, *p*<0.001) perceived risk of getting COVID-19 in the future, and receiving any vaccine in the past 5 years (aOR= 1.6, 95% CI 1.1–2.5, *p*=0.017). However, participants who were reluctant or hesitant to get vaccination before (aOR= 0.6, 95% CI 0.4–0.9, *p*=0.036) were less likely to take up the COVID-19 vaccine (Table 4).

**Table 4:** A multivariable logistic regression showing factors associated with acceptability of the COVID-19 vaccine among medical students in Uganda

Variables	Adjusted odds ratio (aOR)	95% CI	***p***-value
**Sex**			
Female	Reference		
Male	1.9	1.3–2.9	0.001
**Marital status**			
Married	Reference		
Separated	1.9	0.1–38.1	0.675
Single	2.1	1.1–3.9	0.022
**Religion**			
Anglican	Reference		
Muslim	0.9	0.5–1.5	0.61
Other	1.1	0.5–2.5	0.819
Pentecostal	0.5	0.3–1.0	0.042
Roman Catholic	0.8	0.5–1.3	0.294
SDA	1.6	0.6–4.4	0.356
**How likely do you think you will get COVID-19 in future?**			
Not at all	Reference		
Slightly	1.5	0.9–2.6	0.153
Moderate	2.2	1.2–4.1	0.008
Very likely	3.5	1.7–6.9	<0.001
Extremely likely	2.7	1.0–7.4	0.059
**Overall, how worried are you about coronavirus?**			
Not at all worried	Reference		
Somewhat worried	1.2	0.6–2.6	0.579
Not very worried	0.8	0.4–1.6	0.553
Very worried	1.6	0.7–3.9	0.281
Extremely worried	1.4	0.5–3.7	0.535
**To what extent do you think coronavirus poses a risk to you personally?**			
No risk at all	Reference		
Minor risk	0.9	0.3–2.3	0.815
Moderate risk	1.0	0.3–2.7	0.931
Major risk	0.6	0.2–1.9	0.432
**Do you think coronavirus poses a risk to people in Uganda?**			
No risk at all	Reference		
Minor risk	1.1	0.3–5.0	0.863
Moderate risk	1.5	0.3–6.8	0.566
Major risk	3.0	0.7–13.7	0.157
**Do you know if you have had, or currently have, coronavirus?**			
I have definitely not had it	Reference		
I think I have probably not had it	0.8	0.4–1.7	0.539
I think I have probably had it	1.5	0.7–3.3	0.333
I have definitely had it	1.0	0.5–2.3	0.948
**I think I have some immunity to coronavirus**			
Strongly disagree	Reference		
Disagree	0.8	0.3–2.3	0.706
Neutral	0.5	0.2–1.4	0.185
Agree	0.8	0.3–2.2	0.716
Strongly agree	0.9	0.3–2.4	0.828
**Have you been vaccinated before in the past 5 years?**			
No	Reference		
Yes	1.6	1.1–2.5	0.017
**Have you ever been reluctant or hesitate to get a vaccination before?**			
No	Reference		
Yes	0.6	0.4–0.9	0.036

### Vaccine hesitancy among medical students

About two third (66.8%, *n*=401) of the participants had not received any vaccine in the past 5 years. However, (30.7%, *n*=184) reported having been hesitant. The most alluded to reason for vaccination hesitancy was concern about vaccines safety or their side effects (*n*=78, 19.9%) (Table 5).

**Table 5:** Vaccine hesitancy among medical students in Uganda

Hesitancy	Frequency	%
**Have you been vaccinated before in the past 5 years?**		
Yes	199	33.2
No	401	66.8
**Have you ever been reluctant or hesitate to get a vaccination before?**		
Yes	184	30.7
No	416	69.3
**Reason for hesitancy**		
Did not think the vaccine was safe/concerned about side effects	78	19.9
Did not think it was needed	54	13.8
Did not think the vaccine was effective	42	10.7
Heard or read negative media	41	10.5
Fear of needles	35	9.0
Did not know where to get good/reliable information	34	8.7
Did not know where to get vaccination	31	7.9
Someone else told me that the vaccine was not safe	27	6.9
Someone else told me they had had a bad reaction from the vaccine	19	4.9
Had a bad experience with previous vaccinator/health clinic	5	1.3
Had a bad experience or reaction with previous vaccination	5	1.3
Religious reasons	4	1.0
Other beliefs/traditional medicine	4	1.0
Fear of fake vaccines	4	1.0
Laziness	4	1.0
Not interested	2	0.5
Already had the disease	1	0.3
Costs	1	0.3

### COVID-19 risk perception and testing among medical students

Among the participants, 188(30.5%) perceived a slight risk of getting COVID-19, and 206 (34.3%) were not very worried about the disease. Also, 212 (35.3%) and 211 (35.2%) thought that COVID-19 possesses a minor and moderate risk to them, respectively. Of the 171 (28.5%) participants who tested for COVID-19 before, 29 (4.8%) reported having tested positive. One hundred eighty-four (30.7%) students believed they have acquired immunity against COVID-19 (Table 6).

**Table 6:** COVID-19 risk perception and testing among medical students

Perception	Frequency	%
**How likely do you think you will get COVID-19 in future?**		
Extremely likely	23	3.8
Very likely	86	14.3
Moderate	148	24.7
Slightly	183	30.5
Not at all	160	26.7
**Overall, how worried are you about coronavirus?**		
Extremely worried	37	6.2
Very worried	75	12.5
Not very worried	206	34.3
Somewhat worried	190	31.7
Not at all worried	92	15.3
**To what extent do you think coronavirus poses a risk to you personally?**		
Major risk	132	22.0
Moderate risk	211	35.2
Minor risk	212	35.3
No risk at all	45	7.5
**Do you think coronavirus poses a risk to people in Uganda?**		
Major risk	225	37.5
Moderate risk	240	40.0
Minor risk	118	19.7
No risk at all	17	2.8
**Do you know if you have had, or currently have, coronavirus?**		
I have definitely had it	46	7.7
I have definitely not had it	206	34.3
I think I have probably had it	191	31.8
I think I have probably not had it	157	26.2
**Have you been tested for coronavirus?**		
No	429	71.5
Yes—positive	29	4.8
Yes—negative	142	23.7
**Has any of your family members tested for COVID-19?**		
No	345	57.5
Yes—positive	78	13.0
Yes—negative	177	29.5
**Has any of your friends tested positive for COVID-19?**		
No	253	42.2
Yes—positive	221	36.8
Yes—negative	126	21.0
**I think I have some immunity to coronavirus**		
Strongly agree	160	26.7
Agree	184	30.7
Neutral	171	28.5
Disagree	61	10.2
Strongly disagree	24	4.0

## Discussion

Vaccine hesitancy has been a domain of concern globally for several decades now and the picture is more contentious with the current COVID-19 vaccination due to the infodemic and conspiracies surrounding the disease [14]. In this study, we set out to find the COVID-19 vaccine acceptability, hesitancy, and associated factors among medical students in Uganda. To our knowledge, this is the first study of its kind in Uganda and the African continent at large to examine acceptance and hesitancy towards the COVID-19 vaccine among health care students.

Firstly, our study reveals that only 37.3 % of Ugandan medical students are willing to take up the COVID-19 vaccine. This acceptance level is slightly higher than reported among Egyptian medical students (35%) [18]. Acceptance levels are much higher among students from Italy (86.1%) [12], South Carolina (60.6%) [1], and nursing students (43.8%) across seven countries [19]. The most cited reasons for acceptance of the COVID-19 vaccine were protecting self and others from COVID-19 similar to a study among Egyptian medical students [18]. This finding is supported by Brewer et al. who reported that anticipated regret for lack of action (i.e., not getting a vaccination and being infected and/or infecting loved ones) is correlated with a higher likelihood of vaccination [20].This study reveals that males are twice more likely to take up the COVID-19 vaccine than their female counterparts, a finding that has been reported by other studies [19, 21]. Our earlier study among Ugandan medical students showed higher negative attitudes among females towards COVID-19 which further underscores this finding [16].

Secondly, we found that 30.7% of the medical students were hesitant about the COVID-19 vaccination. Hesitancy towards COVID-19 vaccination among university students has been reported elsewhere. Our findings are much lower than reported among Egyptian medical students (46%) [18]; however, relatively similar findings were reported among medical students in Malta (30.5%) [11], slightly higher than hesitancy among medical students from South Carolina (24.3%) [1], and Michigan (23%) [13], and way higher than that reported among medical students in Italy (13.9%) [12] and India (10.6%) [22].This discrepancy could be explained by the variable impact of COVID-19 across the globe with a less severe form of the disease and cases in Africa and Uganda in particular. This could directly affect individuals’ risk perception of COVID-19 and undermine their decision to take up the vaccine.

In a multicenter study, Evridiki et al. reported that increased risk perception towards COVID-19 was associated with the likely uptake of the COVID-19 vaccine [19].Indeed our results show that the highest proportion (30.5%) of students perceived a slight risk of getting COVID-19 in future, and 34.3% were not worried about the disease and it is surely not surprising that uptake was likely among participants that perceived high risk of getting COVID-19 in the future. The most given reason for hesitancy towards the COVID-19 vaccine in this study was concern about its safety and side effects as similarly reported in various other studies [13, 18, 19, 22].

Medical or health care students are thought to be a medically updated and insightful population that would readily take up the vaccine which is paradoxically unlikely. Health care professionals have also been relied on to influence decisions of the general public who seek information from them towards the uptake of vaccines [23, 24]. Therefore in such a situation where they are hesitant warrants more public campaigns and advocacy engaging, all people irrespective of their medical knowledge background on the safety and importance of this vaccination.

The pandemic has been surrounded by a lot of conspiracies that could have greatly swayed many people into hesitancy. From our study having heard negative information about the vaccine and its side effects ranked high among reasons for hesitancy. Furthermore, social media was reported as the major source of negative information about the COVID-19 vaccine. Indeed, Sallam et al. [21] reported that respondents who did not rely on social media as their source of information were likely to accept the vaccine similar to Saied et al. [18] who showed that the hesitancy group reported social media as their major source of COVID-19 information. It is therefore imperative that medical students are encouraged to rely more on other sources of information with censored information than social media.

## Limitations

One of the limitations in this study was the unequal distribution of respondents from the different medical schools and the relatively low sample size compared to the total number of students in these universities. This is due to the difference in the total number of students with newer universities having fewer students and the low response to online studies, especially that it was not incentivized. Sampling bias due to convenience sampling used in the study limits the representativeness of the study. Self-selection bias may also have occurred due to some potential respondents not having Internet access and thus not being aware of the existence of the survey.

## Strengths of the study

The study provides results from a large cross-section of students in 10 different universities and variable programs; therefore, the results can be generalized. Sending daily reminders to the eligible participants on the targeted WhatsApp groups lessened possible response bias associated with online surveys.

## Future directions/research

A qualitative research study involving a larger sample size to dig deeper into the sentiments of both medical and non-medical students about the COVID-19 vaccine could provide more precise information for targeted messages towards demystifying and changing the attitude of this group of the population towards COVID-19 vaccination.

## Conclusion

In conclusion, this study has shown high low levels of acceptance towards COVID-19 vaccine among medical students which poses an evident risk on the battle towards the COVID-19 in the future especially when we are seeing third waves in some countries. There is a lot of complacency towards COVID-19 with low perceived risks among medical students in Uganda and the majority has been corrupted by the negative information on social media that has swayed them into hesitating vaccination. Much effort needs to be geared towards encouraging medical students to take up the vaccine and providing information about the safety and effectiveness of these vaccines

## Data Availability

All data generated or analyzed during the current study is not publicly available due to some individualized information it contains but are available from the corresponding author on reasonable request.

## Abbreviations

COVID-19: Coronavirus disease-2019
SARS-CoV-2: Severe acute respiratory syndrome-coronavirus-2
WHO: World Health Organization
COVAX: Coronavirus disease-2019 Vaccines Global Access
MBChB: Bachelor of Medicine and Bachelor of Surgery
BDS: Bachelor of Dental Surgery
BNS: Bachelor of Nursing Science
BNA: Bachelor of Anesthesia
BPHARM: Bachelor of Pharmacy
BLT: Bachelor of Biomedical Laboratory Technology
BBS: Bachelors of Biomedical Sciences
